# Transcranial doppler detected right-to-left shunt is common but not associated with MRI white matter hyperintensity burden: a cross-sectional study

**DOI:** 10.1093/esj/aakaf029

**Published:** 2026-01-01

**Authors:** Francesco Fisicaro, Mariagiovanna Cantone, Klizia Cortese, Raffaele Ferri, Giuseppe Lanza, Christian Messina, Manuela Pennisi, Marialuisa Zedde, Mario Zappia, Rita Bella

**Affiliations:** Primary Health Care Unit, Provincial Health Authority of Siracusa, Siracusa, Italy; Unit of Neurology, Policlinico University Hospital “G. Rodolico-San Marco”, Catania, Italy; Department of Educational Sciences, University of Catania, Catania, Italy; Clinical Neurophysiology Research Unit, Oasi Research Institute-IRCCS, Troina, Italy; Clinical Neurophysiology Research Unit, Oasi Research Institute-IRCCS, Troina, Italy; Department of Surgery and Medical-Surgical Specialties, University of Catania, Catania, Italy; Primary Health Care Unit, Provincial Health Authority of Catania, Catania, Italy; Department of Biomedical and Biotechnological Sciences, University of Catania, Catania, Italy; Neurology Unit, Stroke Unit, Azienda Unità Sanitaria Locale-IRCCS di Reggio Emilia, Reggio Emilia, Italy; Department of Medical and Surgical Sciences and Advanced Technologies “G. F. Ingrassia”, University of Catania, Catania, Italy; Department of Medical and Surgical Sciences and Advanced Technologies “G. F. Ingrassia”, University of Catania, Catania, Italy

**Keywords:** magnetic resonance imaging, prevalence, right-to-left shunt, transcranial doppler sonography, white matter hyperintensities

## Abstract

**Introduction:**

Right-to-left shunt (RLS) associated with a patent foramen ovale has been related with ischemic stroke. However, its relationship with MRI white matter hyperintensities (WMHs) remains debated. This cross-sectional, single-centre study investigated the prevalence of RLS detected by transcranial Doppler sonography (TCD) and its association with vascular lesions on MRI.

**Patients and methods:**

502 outpatients (mean age 47.8 ± 13 years; 45% male) with non-specific neurological symptoms underwent brain MRI and TCD with contrast saline. WMH severity was visually graded using the Fazekas scale.

**Results:**

RLS was detected in 39% of the sample. No difference was found in demographics and clinical variables between those with and without RLS. No association was also found between RLS and MRI lesion load. As expected, a significant (*P* < .001) positive correlation was identified between age and Fazekas scores (ie, higher scores with increasing age). No effect on lesion load was found for sex, hypercholesterolemia, diabetes, obesity and smoking, while a statistically significant association (*P* = .016) was present for arterial hypertension (odds ratio 1.68, 95% CI, 1.10–2.56; among those with higher Fazekas scores). Finally, no significant association was found between RLS magnitude, both at rest and during the Valsalva manoeuver and the Fazekas scores.

**Discussion:**

Although RLS was frequently detected in this cohort, it was not associated with the presence or severity of WMHs, which were instead driven by age and arterial hypertension. These findings support WMHs as MRI marker of cerebral small vessel disease rather than subclinical paradoxical embolism. This also suggests limited utility of routine TCD screening for RLS in patients with incidental WMHs and no history or sign of embolic features.

**Conclusions:**

In patients with non-specific neurological symptoms, we detected a high occurrence of RLS, although this was not associated with an increased risk or severity of WMHs. As such, paradoxical embolism may not be a major determinant of subclinical WMHs in this population.

## Graphical Abstract

**Graphical abstract:**
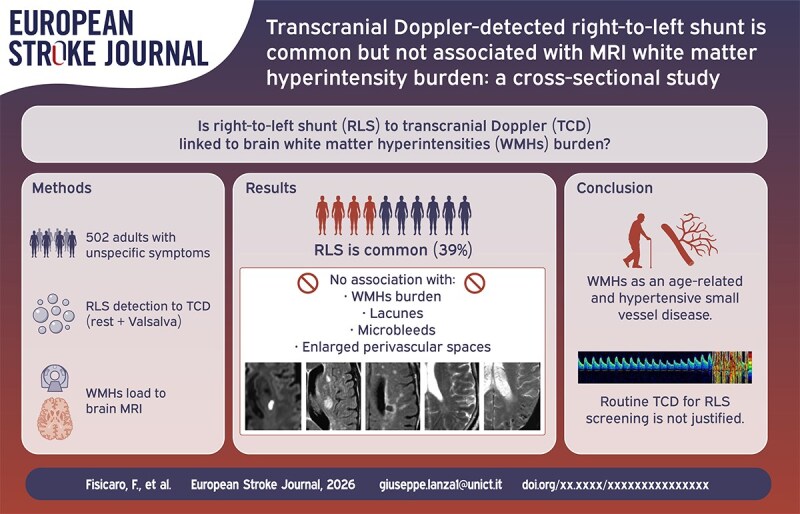

## Introduction

Right-to-left circulatory shunt (RLS) in adults is most commonly associated with a patent foramen ovale (PFO), which is a congenital heart anatomic variant related to the failed closure of the antenatal interatrial communication.^1^^,^^2^ Although most of epidemiological studies on PFO prevalence are dated,^3^ converging evidence shows that PFO occurs very commonly in the healthy adult population, ranging from 15% to 35% in autopsy studies,^4^^,^^5^ from 15% to 25% on transthoracic echocardiography (TTE),^5–8^ from 11% to 43% on transesophageal echocardiography (TEE), and from 16% to 44% on transcranial Doppler sonography (TCD).^5^ Because of its high prevalence in the general population (~1 out of 4 healthy subjects), the etiological role of PFO in vascular diseases is controversial.

Nevertheless, an association between PFO and ischemic stroke has been found in patients with cryptogenic stroke, especially in those younger than 55 years^9–11^ and in those with cortical infarcts, as typically occurs in cardioembolic strokes.^12^ Indeed, PFO allows transient RLS, particularly during the Valsalva manoeuver (VM) or in cases of elevated right atrial pressure, which can facilitate paradoxical embolism by enabling venous thrombi or microemboli to bypass the pulmonary circulation, eventually reaching the cerebral vasculature, among others.^13^ However, therapeutic indications, including its closure or not, are still debated in cryptogenic stroke.^14^^,^^15^

According to some cohort studies, the prevalence of PFO is higher in patients with migraine with aura^16^^,^^17^ and in those with stroke and migraine,^5^^,^^18^ but also in those with vascular cognitive disorders.^19–23^ In this context, the neuroradiological evidence of subclinical lesions, most typically located in the deep white matter and in the posterior artery territories in patients with migraine, opens intriguing pathogenic views.^24^ Additionally, RLS may cause platypnea-orthodeoxia syndrome and be associated with clinical outcomes in patients with obstructive sleep apnea syndrome (OSAS) or chronic obstructive pulmonary disease.^25–27^ Hypercoagulable states, in particular due to the prothrombin G20210A mutation and the Factor V Leiden, are recognised as additional risk factors for cryptogenic stroke in patients with PFO.^28^^,^^29^ More recently, some cohort studies found that PFO may be associated also with white matter hyperintensities (WMHs) on brain MRI, which expose at higher risk of major cerebrovascular events and cognitive decline, especially in those with arterial hypertension or atrial fibrillation.^30^^,^^31^

In this scenario, TCD is a widely available, non-invasive and reliable tool for screening and monitoring RLS, which should be further investigated with a TEE to identify high-risk PFO-related features. However, compared to TEE, the sensitivity and specificity of aerated-saline solution TCD for PFO are 94% and 92%, respectively, according to a meta-analysis included in the European position paper on PFO management.^32^ Therefore, TCD can be reliably applied for the detection of intracardiac RLS, mostly due to PFO, thus identifying a potential embolic source to the brain and allowing the quantification of the shunt magnitude as well.^5^^,^^33^

In addition to these established clinical indications for TCD, in “real world” clinical settings, patients with generic or subjective neurological symptoms, as well as those with thrombophilia-related mutations, usually undergo brain MRI and, if unspecific gliosis or multiple small cortical infarcts are detected, a subsequent TCD to screen them for PFO is usually performed.^34^ In this context, it should be acknowledged that a pathophysiological link between PFO and WMHs or small cortical infarcts via paradoxical microembolism, endothelial dysfunction and impaired cerebral microcirculation has been proposed and explained by the fact that the pulmonary filter bypass allows not only the passage of visible emboli but also microparticles, vasoactive substances and other inflammatory mediators till the cerebral microcirculation.^18^ Nevertheless, as clearly defined in the Standards for Reporting Vascular Changes on Neuroimaging (STRIVE 1.0) and updated STRIVE 2.0 consensus criteria, WMHs should be interpreted primarily as small vessel pathology,^35^^,^^36^ a concept reinforced by the ESO guidelines on covert small vessel disease.^37^ As such, WMHs are often discovered as “incidental” on clinical reads, in the absence of any specific clinical syndrome.

In the present study, a large cohort of patients who underwent brain MRI for non-specific neurological symptoms were screened for RLS in a qualified TCD center. We aimed to assess the prevalence of RLS in this population and the correlation between RLS magnitude and MRI lesion load. We hypothesised that patients with RLS might have a higher risk or load of WMHs compared to those without. Additionally, we evaluated the correlation between RLS and any further imaging features according to the STRIVE 1.0.^35^

## Patients and methods

### Study design

**Figure 1:**
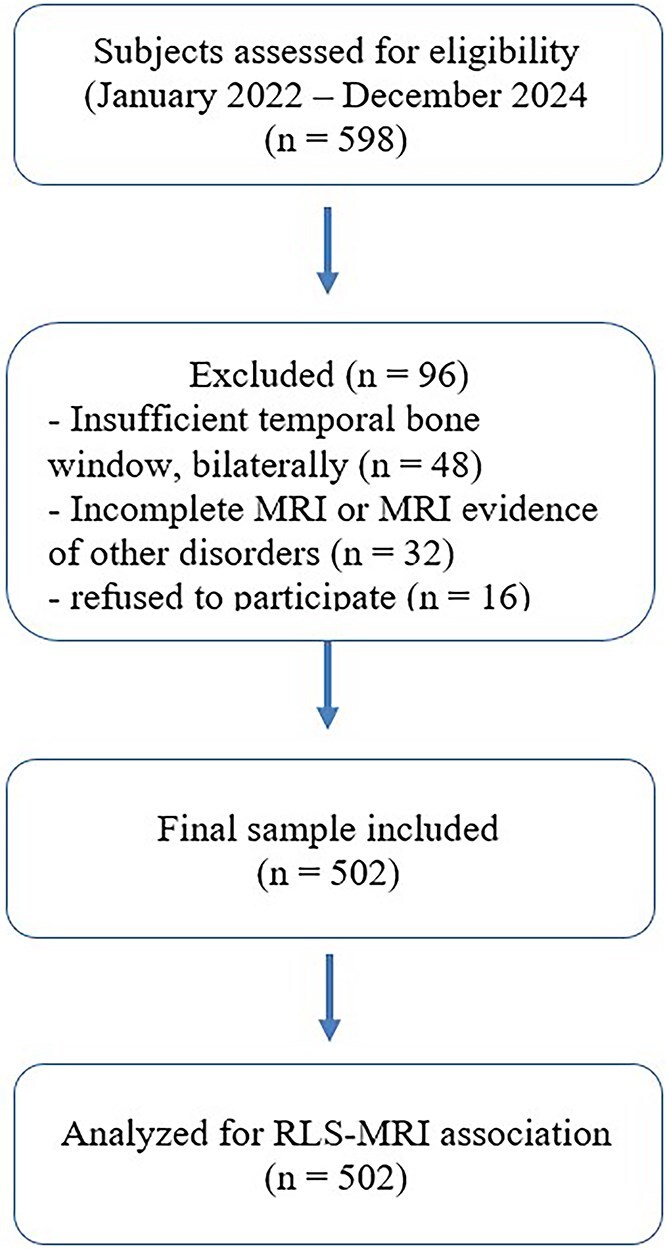
Flow diagram illustrating participant inclusion and exclusion process in the cross-sectional study.

This cross-sectional single-centre Italian study (2022–2024) investigated the prevalence of RLS detected by TCD and its association with the vascular lesion burden on brain MRI. Cross-sectional outpatient cohort adhered to the STROBE reporting guidelines (see Supplementary material), whereas a detailed inclusion/exclusion flow diagram is provided in Figure 1.

### Participants and assessment

A total of 502 subjects, ranging from 18 to 79 years old and referred to the Cerebrovascular Diseases Center of the Azienda Ospedaliera Universitaria Policlinico “G. Rodolico-San Marco” of Catania (Italy), were consecutively recruited from January 2022 to December 2024. A group of 96 subjects was excluded for different reasons, including: a lack of or insufficient temporal bone window, bilaterally; an incomplete MRI exam or MRI evidence of other disorders; refuse to participate. All participants were referred for TCD evaluation by their general practitioners or other specialists (mostly cardiologists) because of one or more unspecific neurological symptoms (eg, dizziness, instability, headache, diffuse sensory disturbances) or for the previous detection of a thrombophilia-associated mutation, along with the neuroradiological evidence of WMHs or other STRIVE 1.0 features,^36^ as well as in cases of diagnosed or suspected PFO on TTE. Subjects were excluded if they had a history of any other neurological disease, including stroke or transient ischemic attack, migraine with aura, tumours or any systemic disease associated with cerebrovascular involvement.

Before being referred to the TCD center, all of them underwent a 2D-brain 1.5 T-MRI, which included T1-, T2-, proton density-weighted and fluid-attenuated inversion recovery scans, in three projections (coronal, axial, sagittal); slice thickness was 5 mm, with a 0.5 mm slice gap. Only MRI scans that met the minimum criteria suggested by the STRIVE 1.0 criteria were selected, in order to ensure uniformity of the investigation of the exams and interpretation of the findings.^35^ The severity of WMHs was graded according to the visual scale score of Fazekas: 0 = absence; 1 = punctuate foci; 2 = partially confluent foci; 3 = large confluent areas. All MRI images provided by the patients were collected and visually inspected by a trained neuroradiologist.^38^

Each participant underwent a complete clinical-demographic assessment and a full neurological examination. Clinical history, including risk factors for cardio- and cerebrovascular diseases, ie, age, sex, obesity, diabetes, arterial hypertension, hypercholesterolemia, atrial fibrillation, smoking habit, thrombop-hilia-related mutations (eg, factor V_G1691A_ mutation, prothrombin _G20210A_ variant and TT_677_ genotype of methylenetetrahydrofolate reductase), were recorded.

The study was carried out in accordance with the Declaration of Helsinki of 1964 and its later amendments. The protocol was approved by the Ethics Committee of the Azienda Ospedaliero-Universitaria Policlinico “G. Rodolico-San Marco” of Catania (protocol code: 292/prot. n. 871) and all participants gave their written informed consent prior to entry.

### Transcranial doppler sonography

Transcranial Doppler sonography was performed with Compumedics DWL equipment, Multi-Dop X digital, Singen (Germany). All examinations were performed by the same expert operator (R.B.), who remained “blind” with respect to the patients’ clinical status and MRI features. Practically, the TCD ultrasound probe was placed over the temporal window to optimally insonate the middle cerebral artery (MCA). Right-to-left shunt detection involved the recording of agitated saline mimicking microemboli, passing through the MCA during both normal respiration and after the VM, as a series of different severity of embolic tracks seen on the ultrasonography screen. Shunt quantification was then possible by counting the number of microbubble (MB) signals.^5^

All TCD procedures were carried out according to the internationally validated method, described in detail by Angeli et al.^39^ Briefly, it consisted of the injection of 9 mL of previously shaken saline as a contrast-enhancing agent into an antecubital vein while recording the flow velocity of the MCA, insonated through the temporal window on the right or left side at a depth of 50−60 mm, with a handheld probe. The appearance of transient spikes on the velocity spectral curve within 10−40 seconds of the intravenous injection is deemed positive. The same procedure was subsequently repeated during the VM. A positive test was defined by the number of MB that were seen and classified as follows: negative test (no MB), mild grade shunt (1−10 MB), moderate grade shunt (>10 MB) and high grade shunt (“shower” effect or “curtain” effect), according to the case–control study by Serena et al.,^40^ which differs from the ICC/Venice grading system.^41^ Regarding the pathophysiological features of the shunt, it was defined as permanent when detected at rest and latent when detected only during VM. Blood pressure and heart rate were recorded before each examination. Data were collected on a dedicated PC and stored in an ad hoc database.

### Statistical analysis

Statistical analysis was performed with the Jamovi software.^42^ Descriptive statistics for numerical (mean, standard deviation and range) and categorical (frequencies) variables were computed for the demographics and clinical features of the enrolled subjects. Univariable analyses, utilising linear model ANOVA and Pearson’s Chi-squared tests, were conducted to compare the patients’ characteristics with and without RLS. Association measures, ie, odds ratio (OR) and relative risk (RR), were also computed between RLS exposure and MRI abnormalities, according to the STRIVE 1.0 criteria. To evaluate any correlation between the MRI lesion’s load and RLS magnitude, we used a generalised model performing an ordinal logistic regression with the Fazekas white matter score as the dependent variable, the RLS magnitude at rest and during the VM as factors and age as a covariate. The effect of other clinical and demographic variables on MRI lesions was also evaluated with the same model. All the variables included in the ordinal logistic regression analysis were tested for violations of the proportional odds assumption with a test of parallel lines. Finally, a binomial logistic regression model with the same parameters was used to evaluate any association between RLS and both deep and superficial enlarged perivascular spaces. A *P*-value lower than .05 was considered as statistically significant.

## Results

A total of 502 subjects were included in the study (226 males, 45% of the sample) with a mean age of 47.8 years (SD 13 years). RLS presence was detected in 39% of the sample (196 subjects). Table 1 shows the characteristics of the sample, divided by RLS status. No difference was found in demographics and clinical variables between patients with RLS and those without. In particular, no association was present between RLS and WMHs (Chi-squared = 0.02, *P* = .864), with an OR of 1.05 (95% CI, 0.63−1.73) and a RR of 1.04 (95% CI, 0.76−1.59). Similarly, no association was found between RLS and enlarged perivascular spaces, cerebral microbleeds or lacunar lesions.

**Table 1:** Demographic and clinical characteristics of the sample, divided by shunt status

	**No shunt** **(*n* = 306)**	**Shunt** **(*n* = 196)**	**Total** **(*n* = 502)**	***P*-value **
**Age**				.207^a^
Mean (SD)	48.4 (13.3)	46.9 (12.5)	47.8 (13.0)	
Range	18.0—79.0	19.0—79.0	18.0—79.0	
**Sex**				.214^b^
Male	131 (42.8%)	95 (48.5%)	226 (45.0%)	
Female	175 (57.2%)	101 (51.5%)	276 (55.0%)	
**WMHs**				.864^b^
	259 (84.6%)	167 (85.2%)	426 (84.9%)	
**Microbleeds**				.176^b^
	6 (2.0%)	1 (0.5%)	7 (1.4%)	
**Enlarged perivascular spaces**				.830^b^
	34 (11.1%)	23 (11.7%)	57 (11.4%)	
**Lacunar lesions**				.698^b^
	40 (13.1%)	28 (14.3%)	68 (13.5%)	
**Arterial hypertension**				.358^b^
	109 (35.6%)	62 (31.6%)	171 (34.1%)	
**Hypercholesterolemia**				.845^b^
	71 (23.2%)	44 (22.4%)	115 (22.9%)	
**Diabetes**				.782^b^
	32 (10.5%)	19 (9.7%)	51 (10.2%)	
**Obesity**				.618^b^
	36 (11.8%)	26 (13.3%)	62 (12.4%)	
**Atrial fibrillation**				.418^b^
	8 (2.6%)	3 (1.5%)	11 (2.2%)	
**Smoking**				.795^b^
	67 (21.9%)	41 (20.9%)	108 (21.5%)	
**Shunt size (rest)**				
No shunt		62 (31.6%)		
Low-grade		57 (29.1%)		
Medium-grade		40 (20.4%)		
High-grade		37 (18.9%)		
**Shunt size (Valsalva)**				
Low-grade		65 (33.2%)		
Medium-grade		29 (14.8%)		
High-grade		102 (52.0%)		

^a^Linear model ANOVA.

^b^Pearson’s Chi-squared test.

Abbreviation: WMHs = white matter hyperintensities.

Tables 2 and 3 show the results of the ordinal regression model. A significant (*P* < .001) positive correlation was found between age and Fazekas scores (ie, higher scores with increasing age). No effect on the lesion load was found for sex, hypercholesterolemia, diabetes, obesity and smoking habits, while a significant (*P* = .016) association was detected for arterial hypertension (OR 1.68 with higher Fazekas scores compared to patients without hypertension). Atrial fibrillation was excluded from the analysis due to the small number of positive patients (11 subjects). Similarly, the effect of thrombophilia-related mutations was not further investigated, given that only nine heterozygous FV_G1691A_, two heterozygous PT_G20210A_ variant and 36 TT_677_ MTHFR (14 homozygous, 22 heterozygous) patients only were present in the sample. No significant association between shunt size at rest and during the VM and Fazekas scores was found. The binomial logistic regression model showed no correlation between RLS size and enlarged perivascular spaces (Table 4). However, a significant positive correlation was found with age (*P* = .003) and arterial hypertension (*P* = .038). Given the small number of cerebral microbleeds, no further analysis was performed on this outcome.

**Table 2:** Ordinal logistic regression model for the Fazekas deep white matter score: Omnibus tests

	***X*** ^**2**^	**df**	***P*-value **
Age	38.602	1	**<.001**
Sex	0.647	1	.421
Shunt size (rest)	6.764	3	.080
Shunt size (Valsalva)	6.367	3	.095
Arterial hypertension	5.835	1	**.016**
Hypercholesterolemia	0.776	1	.378
Diabetes	1.992	1	.158
Obesity	0.141	1	.707
Smoking	0.321	1	.571

Numbers in bold refer to statistically significant *p* values.

**Table 3:** Ordinal logistic regression model for the Fazekas deep white matter score: parameter estimates

					**Exp (B) 95% confidence intervals**			
**Name**	**Effect**	**Estimate**	**SE**	**Exp (B)**	**Lower**	**Upper**	***z***	***P*-value**
(Threshold)	0|1	−1.881	0.249	0.152	0.093	0.249	−7.533	<.001
(Threshold)	1|2	0.593	0.238	1.810	1.135	2.886	2.492	.013
(Threshold)	2|3	1.792	0.252	6.003	3.656	9.855	7.086	<.001
Age	Age	0.051	0.008	1.053	1.036	1.071	6.213	<.001
Sex1	Female—male	0.141	0.176	1.152	0.815	1.628	0.804	.421
Shunt size (rest)1	Low-grade—No	0.244	0.360	1.277	0.630	2.586	0.678	.498
Shunt size (rest)2	Medium-grade—No	0.031	0.465	1.032	0.414	2.568	0.067	.946
Shunt size (rest)3	High-grade—No	1.082	0.491	2.953	1.126	7.742	2.201	.028
Shunt size (Valsalva)1	Low-grade—No	−0.129	0.303	0.879	0.485	1.592	−0.425	.670
Shunt size (Valsalva)2	Medium-grade—No	0.128	0.452	1.138	0.468	2.761	0.284	.776
Shunt size (Valsalva)3	High-grade—No	−0.839	0.393	0.432	0.199	0.933	−2.135	.033
Arterial hypertension1	Yes—No	0.518	0.214	1.680	1.102	2.558	2.415	.016
Hypercholesterolemia1	Yes—No	−0.189	0.215	0.827	0.542	1.262	−0.880	.378
Diabetes1	Yes—No	0.418	0.296	1.520	0.849	2.720	1.411	.158
Obesity1	Yes—No	0.098	0.262	1.104	0.659	1.848	0.375	.707
Smoking1	Yes—No	−0.121	0.215	0.885	0.580	1.349	−0.566	.571

**Table 4:** Binomial logistic regression for the enlarged perivascular spaces

**Model coefficients—enlarged perivascular spaces**							
		**95% Confidence interval**					
**Predictor**	**Estimate**	**Lower**	**Upper**	**SE**	***Z***	***P*-value**	**Odds ratio**
Intercept	−4.645	−6.326	−2.965	0.857	−5.418	<.001	0.009
Age	0.046	0.015	0.077	0.015	2.935	**.003**	1.047
Sex							
Female – Male	−0.508	−1.116	0.098	0.309	−1.642	.100	0.601
Arterial hypertension							
Yes – No	0.726	0.038	1.414	0.351	2.069	**.038**	2.068
Hypercholesterolemia							
Yes – No	−0.167	−0.851	0.515	0.348	−0.481	.630	0.845
Diabetes							
Yes – No	0.612	−0.175	1.400	0.402	1.523	.128	1.844
Obesity							
Yes – No	−0.068	−0.969	0.831	0.459	−0.149	.881	0.933
Smoking							
Yes – No	0.003	−0.735	0.742	0.377	0.008	.993	1.003
Shunt size (rest)							
Low-grade – No	0.374	−0.772	1.520	0.584	0.639	.522	1.453
Medium-grade – No	−0.839	−2.731	1.052	0.965	−0.869	.385	0.432
High-grade – No	−0.702	−2.387	0.983	0.859	−0.816	.414	0.495
Shunt size (Valsalva)							
Low-grade – No	0.464	−0.509	1.438	0.496	0.934	.350	1.590
Medium-grade – No	−1.035	−3.225	1.154	1.117	−0.926	.354	0.355
High-grade – No	0.391	−0.915	1.697	0.666	0.587	.557	1.479

Estimates represent the log odds of “perivascular spaces = yes” vs “perivascular spaces = no”. Numbers in bold refer to statistically significant *p* values.

## Discussion

To our knowledge, this is the largest cross-sectional study investigating the prevalence of RLS to TCD in patients with non-specific neurological symptoms and incidental WMHs on brain MRI. The main findings are the high prevalence (39%) of RLS in this population (a result consistent with previous autopsy and echocardiographic studies^4^^,^^5^), the lack of association between the presence or magnitude of RLS and WMH burden and the age and hypertension as significant independent predictors of WMHs severity.^5^ Overall, this confirms that RLS is a frequent occasional finding, especially in outpatient settings, where patients are referred for unspecific neurological symptoms.

Although the rate of RLS we observed in this cohort might appear rather high (39%), it closely approaches that reported in cohorts representing the general population under 55 years of age, particularly when individuals are investigated for non-specific neurological complaints, as in the present study. Notably, Koutroulou et al.^43^ reported a PFO prevalence of 42.7% in the Greek general population, a figure strikingly similar to our finding; in particular, the authors suggested that PFO prevalence may be higher in certain ethnic groups. In this context, it is intriguing to note that our study was conducted in Catania, a geographical area historically belonging to the ancient “Magna Graecia”. A partially shared genetic background between the populations of Southern Italy and Greece might, therefore, represent a plausible explanation for the comparable prevalence rates observed. Although speculative, this hypothesis reinforces the concept that genetic and ethnic factors may contribute to inter-population variability in PFO prevalence and should be taken into account when interpreting epidemiological data on RLS and PFO distribution.

Earlier studies proposed a link between PFO-associated RLS and subclinical brain lesions. For instance, Kim et al.^34^ found that patients with larger RLS exhibited a greater frequency of multiple small cortical infarcts and posterior circulation involvement, which are features both associated with an embolic rather than a chronic hypoperfusive aetiology. Other studies highlighted the potential role of PFO in increasing susceptibility to small embolic events, which may bypass the pulmonary filter and then impact cerebral microcirculation.^18^ However, our findings support the view that in patients without overt embolic events or cryptogenic stroke, the presence of RLS alone is not sufficient to induce chronic microvascular injury, such as WMHs. This aligns with more recent studies suggesting that, in the absence of clinical embolic phenomena, PFO represents a bystander rather than a causal factor in WMHs pathogenesis.^19^^,^^32^

Nevertheless, it should be acknowledged that WMHs on brain MRI occur very frequently in neurological practice, including in those subjects with PFO, independently of age, symptom onset and disease duration.^44^ Therefore, as recently reported, the presence of PFO may be a risk factor for the development of two distinct cerebrovascular diseases: stroke and incidental WMHs, each characterised by different imaging patterns and pathophysiological mechanisms.^45^

Importantly, the lack of association between RLS magnitude and WMH severity in our analysis further weakens the hypothesis of a direct pathogenic link. Although shunt severity is associated with an increased risk of paradoxical embolism, our data did not show any relationship with WMH burden. Also, a large shunt was not predictive of high-grade Fazekas scores or an increased number of enlarged perivascular spaces. On the contrary, we confirmed the association between enlarged perivascular spaces and both age and hypertension, a finding consistent with the pathophysiology of small vessel disease. Therefore, according to previous studies,^46^^,^^47^ RLS severity detected in routine TCD exams may not directly relate to embolic load or have any clinical impact unless other predisposing factors, such as hypercoagulability, atrial septal aneurysm or arrhythmia, are also present.^13^^,^^28^^,^^34^ Additionally, while RLS is known to be associated with early onset stroke and has specific spatial lesion patterns, WMHs are more typically indicative of chronic changes in cerebral microcirculation.^48^ Moreover, recent evidence highlights that WMHs represent a multifactorial phenotype, only partially attributable to vascular risk, but nonetheless a powerful predictor of stroke and cognitive decline. Nevertheless, it should be noted that the cross-sectional design of the present study precludes inferences about any temporal relationship between RLS and WMHs development. As such, it cannot be excluded that RLS might contribute to WMH progression over time; therefore, longitudinal studies are needed to confirm or rule out this potential link.^35^

Age and hypertension were confirmed to be the only independent predictors of WMH burden in our regression model. The strong association between age and WMHs is well-documented and reflects cumulative vascular injury, endothelial dysfunction and demyelination processes related to ageing.^5^^,^^30^^,^^31^ Similarly, arterial hypertension is a major known contributor to cerebral small vessel pathology, and the OR of 1.68 for higher Fazekas scores we observed in hypertensive individuals is consistent with previous population-based data.^30^ In contrast, other conventional vascular risk factors, such as diabetes, hypercholesterolemia, obesity and smoking, did not show any significant independent effect on WMH severity. This might be explained by the relatively young age of our sample (mean age 48 years) and the generally low burden of comorbidities. Also, our population may have included individuals with less severe systemic vascular disease, given that patients with previous strokes, migraine with aura or overt cerebrovascular disorders were excluded.^49^

A clinically important implication of our study concerns the prescriptive appropriateness of TCD screening for the detection of RLS in patients with incidentally discovered WMHs but without any clinical and imaging suspicion of embolic events. According to the principles of clinical governance, the healthcare system should minimise inappropriate or irrelevant care and maximise net individual health gain. In this case, while TCD remains an essential diagnostic tool for suspected paradoxical embolism, its prescription in the absence of a history of cryptogenic stroke, migraine with aura or thromboembolic phenomena may be inappropriate in the absence of embolic suspicion, consistent with ESO and position paper recommendations. This reinforces existing guideline recommendations that emphasise the importance of screening based on risk stratification.^14^^,^^15^

Some limitations should be acknowledged. First, although the Fazekas scale score provides a validated and widely used metric for WMH burden, more quantitative assessments (eg, volumetric lesion mapping, diffusion tensor imaging) offer greater sensitivity. Second, the relatively low prevalence of some co-variables (eg, thrombophilia mutations or atrial fibrillation) limited the possibility of exploring their interaction with RLS and lesion load. Similarly, given the small number of microbleeds and TEE available, the study was not powered for these endpoints. Third, although our sonographer was blinded to clinical data, MRI assessment was based on visual inspection only and may be prone to inter-rater variability. Finally, since this was an outpatient population, no information is available on their follow-up, as well as on drug treatments and the degree of pharmacological compensation of the risk factors reported. For the same reason, the role of multiple variables possibly underlying WMHs (eg, genetic factors, severity of vascular risk factors, lipoprotein a level, homocysteine level, migraine, OSAS, etc.) could not be included in the regression analysis, thus potentially limiting the generalizability of the results. Future prospective studies using multimodal imaging techniques and comprehensive vascular profiling, including biomarkers of inflammation, endothelial dysfunction and glioneurovascular unit integrity, are warranted to clarify the potential role of PFO in cerebral small vessel diseases. Studies should also assess the prognostic implications of RLS in patients with degenerative or vascular-related cognitive impairment, where WMHs are highly prevalent and clinically significant.^21^^,^^50^

In conclusion, in this large outpatient cohort, RLS was frequent but showed no association with the presence or severity of MRI WMHs. These findings support that, in patients without embolic indications, paradoxical embolism is unlikely to be a determinant of subclinical small vessel disease. Accordingly, transcranial Doppler screening for RLS may be inappropriate in the absence of embolic suspicion, as aligned with current ESO recommendations.

## Supplementary Material

aakaf029_STROBE_checklist

## Data Availability

De-identified participant data and the raw data are available from the corresponding author upon request.

## Patient and public involvement

Patients were not involved in the design, conduct, reporting or dissemination plans of this research.
