# Supplementary material for: Transcranial doppler detected right-to-left shunt is common but not associated with MRI white matter hyperintensity burden: a cross-sectional study
Source: Eur Stroke J. 2026 Jan 1;11(1):aakaf029. doi: 10.1093/esj/aakaf029 (PMC12866658; doi:10.1093/esj/aakaf029)
Supplement: aakaf029_STROBE_checklist [file aakaf029_strobe_checklist.docx]

**Supplementary material**

**STROBE Checklist (Cross-sectional)**

*Manuscript title:* Transcranial Doppler detected right-to-left shunt is common but not associated with MRI white matter hyperintensity burden: a cross-sectional study

*Study design:* Cross-sectional study

| **Item No.** | **STROBE Recommendation** | **Addressed?** | **Where in manuscript** |
| --- | --- | --- | --- |
| 1 | Title & Abstract | Yes | Title; Structured Abstract |
| 2 | Background/Rationale | Yes | Introduction |
| 3 | Objectives | Yes | Introduction; Abstract |
| 4 | Study design | Yes | Methods – Study design |
| 5 | Setting | Yes | Methods – Participants and assessment |
| 6 | Participants | Yes | Methods – Participants and assessment |
| 7 | Variables | Yes | Methods – definitions |
| 8 | Data sources/measurement | Yes | Methods – TCD; MRI |
| 9 | Bias | Yes | Methods |
| 10 | Study size | Partially | Methods |
| 11 | Quantitative variables | Yes | Methods – Statistics |
| 12 | Statistical methods | Yes | Methods – Statistics |
| 13 | Participants flow | Yes (diagram optional) | Methods/Results |
| 14 | Descriptive data | Yes | Results – Table 1 |
| 15 | Outcome data | Yes | Results |
| 16 | Main results | Yes | Results – Tables 2–4 |
| 17 | Other analyses | Yes | Results |
| 18 | Key results | Yes | Discussion |
| 19 | Limitations | Yes | Discussion |
| 20 | Interpretation | Yes | Discussion |
| 21 | Generalisability | Partially | Discussion |
| 22 | Funding | Yes | Funding; COI |
